# Supplementary material for: 21st-century stagnation in unvegetated sand-sea activity
Source: Nat Commun. 2022 Jun 27;13:3670. doi: 10.1038/s41467-022-31123-8 (PMC9237066; doi:10.1038/s41467-022-31123-8)
Supplement: Supplementary file 1 — Supplementary Information [file 41467_2022_31123_MOESM1_ESM.pdf]

Supplementary Information for “21st-century stagnation in unvegetated sand-sea activity”

Andrew Gunn[1,2] Amy East[3] Douglas J. Jerolmack[2,4,*]

[1]School of Earth Amtosphere and Environment, Monash University, Clayton, Australia
[2]Department of Earth and Environmental Sciences, University of Pennsylvania, Philadelphia, USA
[3]Pacific Coastal & Marine Science Center, United States Geological Survey, Santa Cruz, USA
[4]Department of Mechanical Engineering and Applied Mechanics, University of Pennsylvania, Philadelphia, USA
[*]sediment@sas.upenn.edu

**Name**	**Av. Latitude (°)**	**Av. Longitude (°)**	**Area (km^2)**
Rub Al Khali	20.6	230.8	527163
El Djouf	19.8	173.7	454564
Fachi Bilma	17.6	192.5	224704
Grand Erg Oriental	31.0	187.3	182744
Central Takla Makan	39.2	264.0	168779
Central Great Sand Sea	27.4	205.0	167921
Ergs Iguidi & Chech	26.7	177.1	163100
An Nafud & Ad Dahna	27.6	223.0	119612
Munga-Thirri	-25.0	316.9	101813
Grand Erg Occidental	30.4	180.7	72725
Idehan Ubari	27.2	191.8	63209
Idehan Murzuk	24.9	193.1	57416
Trarza Reion Desert	18.3	165.6	44882
Aoukar	17.7	170.7	44831
Azefal, Akchar & Agneitir	20.6	165.4	32654
Namib Sand Sea	-24.9	195.3	31512
Tengger Desert	38.5	284.3	28723
Badain Jaran Desert	40.4	281.8	28112
East Erg Issaouane	27.5	187.8	27579
Southwest Takla Makan	38.2	259.0	24229
Northwest Takla Makan	39.3	260.0	20310
Zaltan Sand Sea	27.3	200.1	17231
Kumtag Desert	39.8	272.1	16683
East Registan Desert	30.5	245.5	15409
Sinai Negev Erg	30.7	213.2	10884
Ramlat Al Sabatayn	15.5	226.2	10110
East Takla Makan	40.2	269.0	9331
Dakhla Farafra	26.5	208.7	8797
Kharan Desert	28.0	244.5	7884
Wahiba Sands	21.9	238.9	7635
Rig-e Yalan	30.3	239.5	7069
West Registan Desert	29.6	243.0	5544
West Erg Issaouane	26.9	186.7	4854
Rig-e Jenn	34.0	233.7	4506
Hobp Desert	40.5	288.3	4172
Thar Desert	26.6	249.7	4012
Yamma Yamma	-26.8	321.3	3949
Ulan Buh Desert	39.9	286.4	3529
Gran Desierto	31.9	65.9	3169
Cunene Namib Desert	-17.5	192.0	3116
Baia dos Tigres Namib Desert	-16.3	192.0	3059
Karakum Desert	39.1	242.1	2162
Skeleton Coast Dune Field	-19.6	192.9	1900
Algodones	32.9	65.0	593

Table S1: **Sand seas in this study**. Names, centroid latitude, centroid longitude and areas are given in columns for the 45 sand seas analyzed in this study from left to right. Rows in order of descending area as in the horizontal axes of Figures S3, S6 and S7.

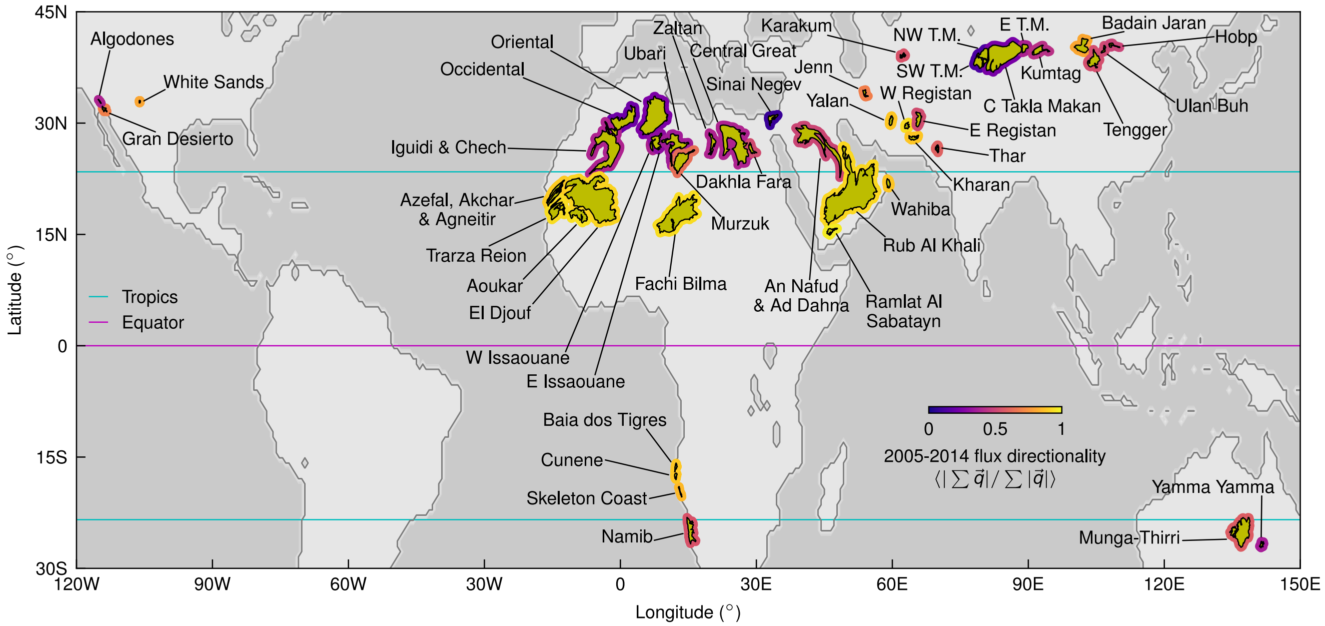

Figure S1: **Annotated map of global sand seas with flux directionality.** A cropped global map showing the sand seas ($n = 45$ yellow with black outlines) analyzed in this study. The sand seas are annotated with abbreviated names (see Table S1 for full names) that omit words in any language like 'desert' or 'dune field' and reduce cardinal directions. Sand seas are outlined with a bold border that is colored by the sand flux directionality over the 2005-2014 period in the 'historical' scenario of the EC-Earth3 ESM. Note the high flux directionality dune fields in tropical Africa and west Asia. The tropics and equator are given, land and ocean are colored by light and dark grey, respectively.

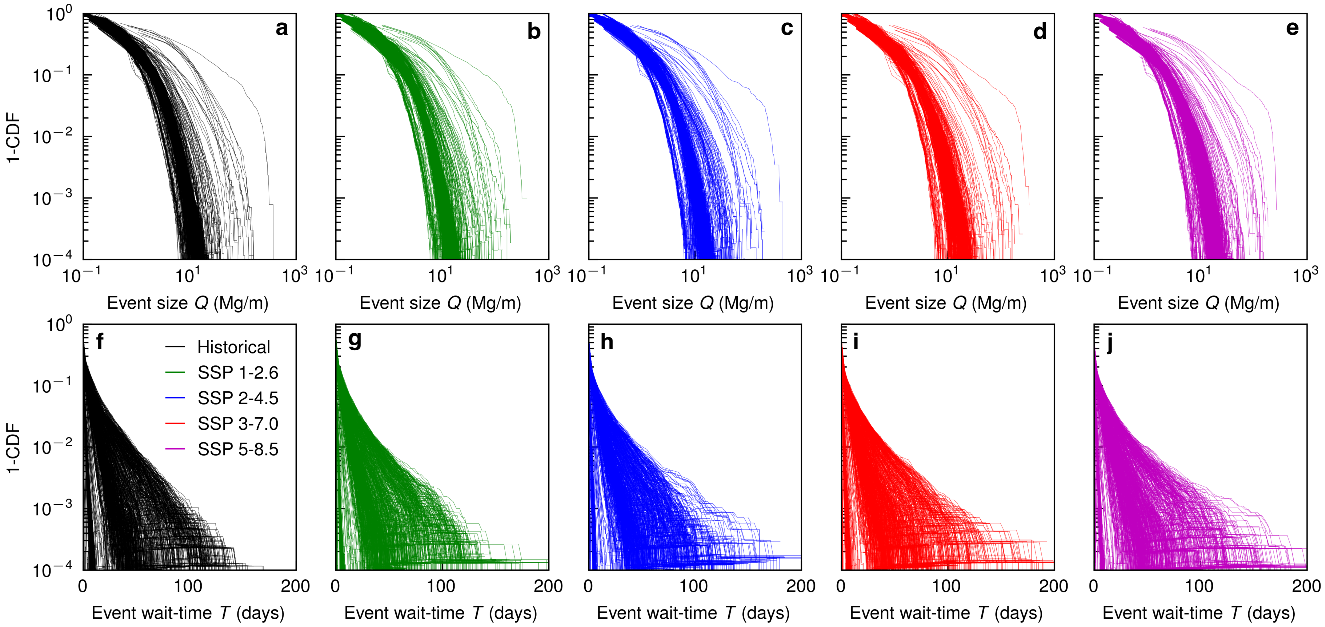

Figure S2: **Frequency-magnitude plots for sand flux events.** EC-Earth3 ESM grid tile 1-CDF plots for sand flux event size Q (Mg/m) (**a**–**e**) and wait-time T (days) (**f**–**j**) in all sand seas for the 2005-2014 historical decade and 2091-2100 future decade in the four tier-1 SSP scenarios (colors given in legend of (f)) in columns. These plots have the logarithmic and linear scales of the horizontal axes in the insets of Figure 2c&d swapped so the distribution shapes can be observed. CDFs are made from the aggregate of ensemble runs.

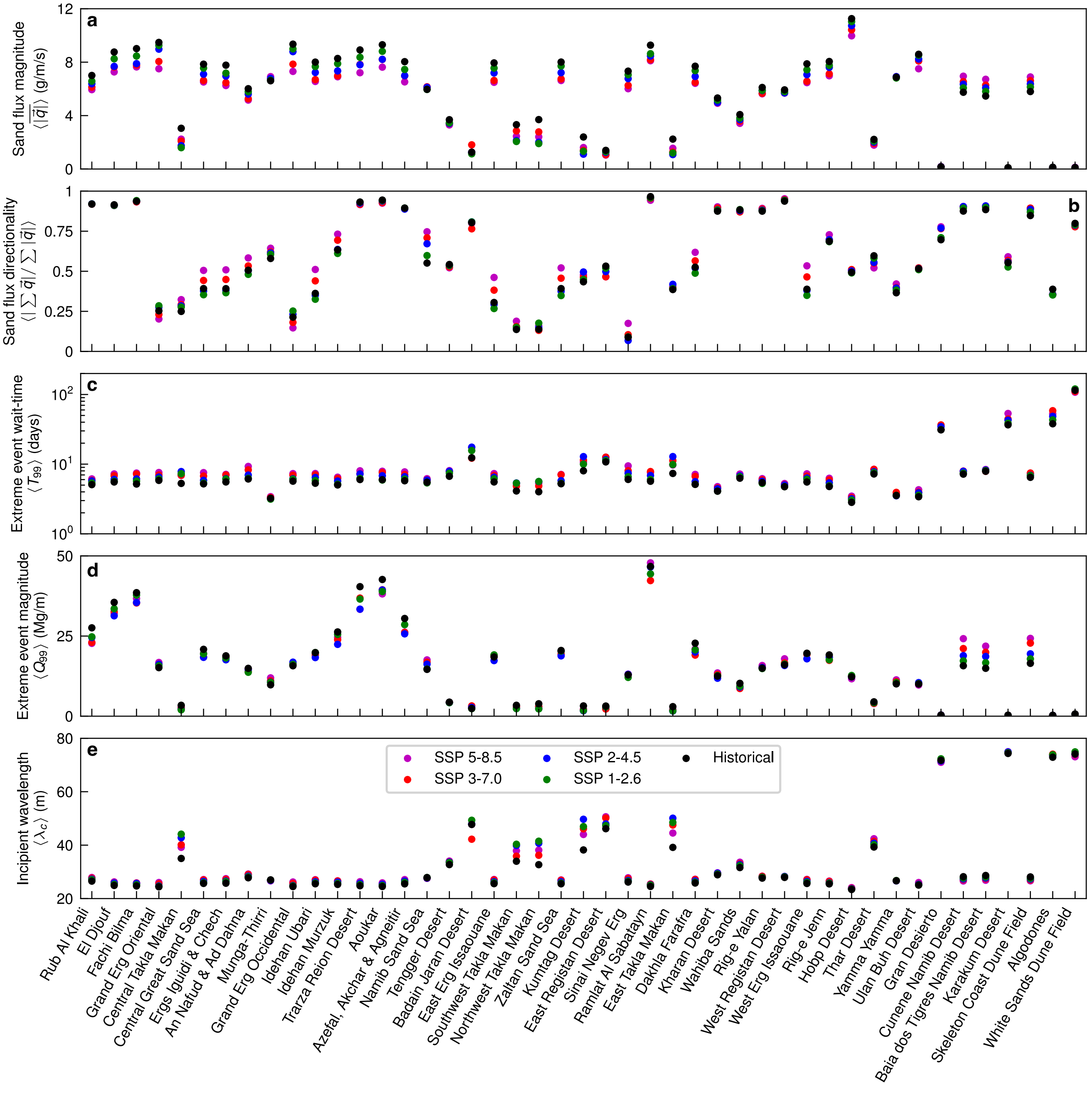

Figure S3: **Absolute decadal averages for key sand-sea variables.** Average (spatially across the sand sea, across ensemble members and in time across the decade) values for each sand sea (organized in descending area order like Table S1 and Figures S6 and S7.) are given for the 2005-2014 historical decade (black) and 2091-2100 future decade in the four tier-1 SSP scenarios (colors given in legend of (e)) for the variables; (**a**) sand flux magnitude $\langle|\overline{\vec{q}}|\rangle$ (g/m/s), (**b**) sand flux directionality $\langle|\sum\vec{q}|/\sum|\vec{q}|\rangle$, (**c**) 99th percentile flux event wait-time $\langle T_{99}\rangle$ (days), (**d**) 99th percentile flux event size $\langle Q_{99}\rangle$ (Mg/m), and (**e**) incipient wavelength $\langle\lambda_c\rangle$ (m).

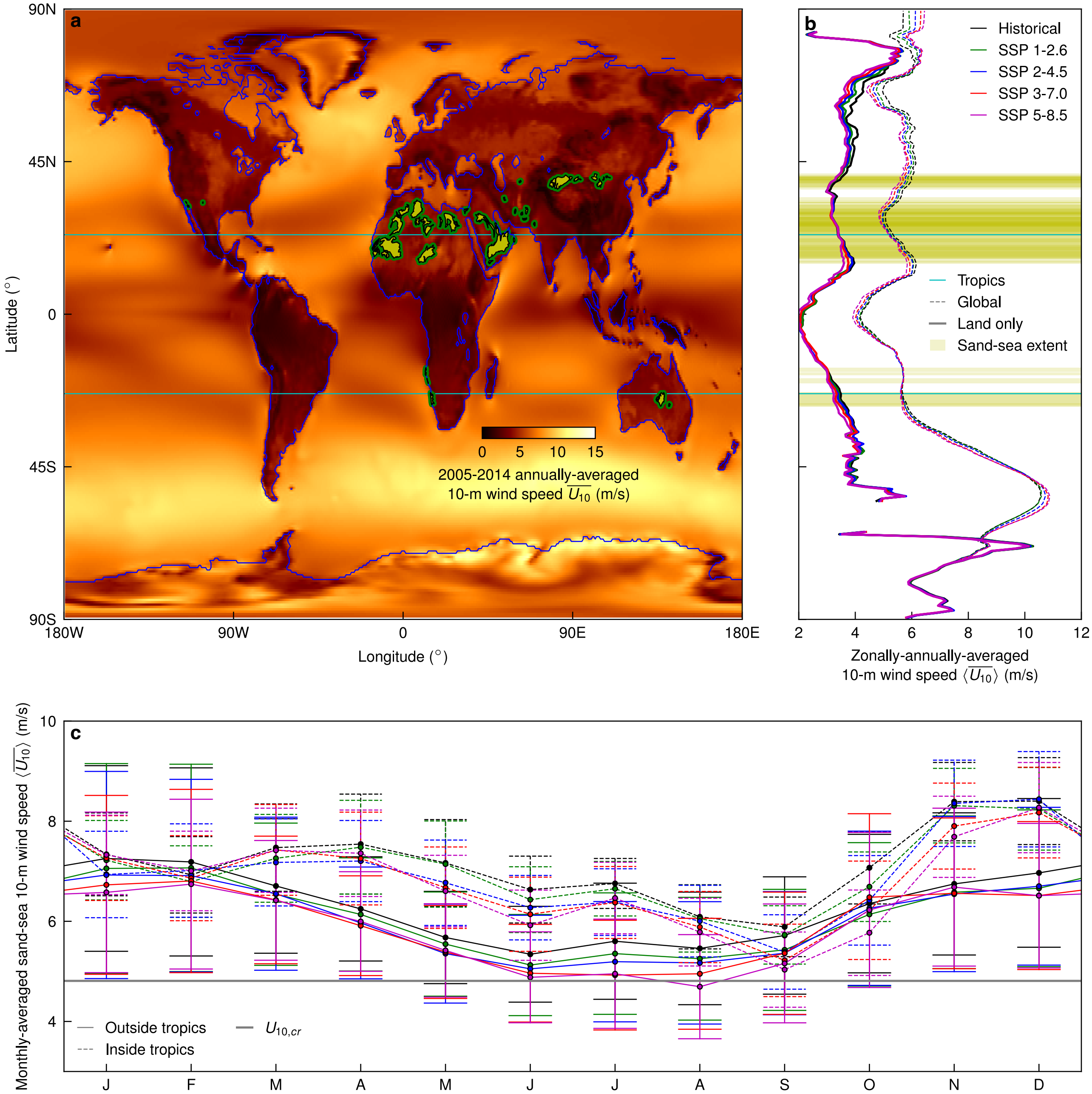

Figure S4: Caption on following page.

Figure S4: **First-order global wind pattern attribution of sand flux changes.** (**a**) A map of average (across ensemble members and in time across the decade) 10-m wind speed for 2005-2014 overlaid by; the sand seas as in Figure 1a, the land-ocean boundary in the EC-Earth3 ESM, and the tropics. (**b**) The zonal-average of the map in (a) (black) with the equivalent for the average (across longitude, ensemble members and in time across the decade) 10-m wind speed for the 20091-2100 decade in the four tier-1 SSP scenarios (colors in top right legend). (b) has the global zonal-average (dashed lines) and land-only zonal-average (thick lines) with the tropics noted and the latitudinal extents of all sand seas shaded. In (b) the increase in strength and latitude of the Southern Annular Mode is evident, as is the weakening of the trade winds in both hemispheres. Subtropical sand seas exist in the zonal-minima of wind speed between the Westerlies and Trade Winds. (**c**) The average (across ensemble members and in time across the decade) monthly-averaged 10-m wind speed climatology for sand sea tiles within (dashed) and outside (solid) the tropics to show how the persistence and strength of trade winds maintains more consistent and higher sand flux throughout the year for sand seas within the tropics. Climatologies given for the 2005-2014 historical decade (black) to the 2091-2100 future decade in the four Tier-1 scenarios (colored, shown in top right legend of (b)). The grey line is the threshold 10-m wind speed in order to move sand (note these wind speeds are time averages), and the error bars are from the ensemble.

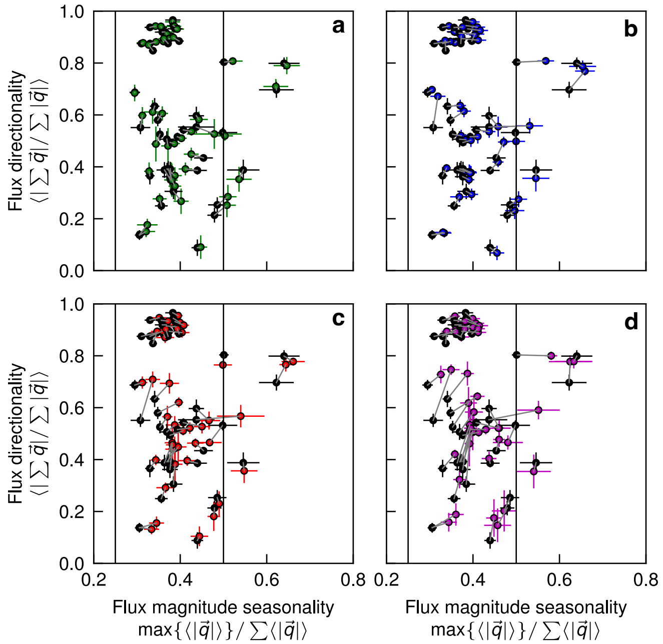

Figure S5: **Linkage of seasonality and directionality in sand flux.** Changes in average (spatially across the sand sea, across ensemble members and in time across the decade) flux magnitude seasonality $\max\{\langle|\vec{q}|\rangle\}/\sum\langle|\vec{q}|\rangle$ and flux directionality $\langle|\sum\vec{q}|/\sum|\vec{q}|\rangle$ from the 2005-2014 historical decade (black dots) to the 2091-2100 future decade in the four tier-1 scenarios (colored dots); (**a**) SSP1-2.6 (green), (**b**) SSP2-4.5 (blue), (**c**) SSP3-7.0 (red), (**d**) SSP5-8.5 (purple). Sand flux magnitude seasonality is the proportion of the annual sand flux magnitude $\sum\langle|\vec{q}|\rangle$ (kg/m/s) that occurs in the quarter of the year (3-month consecutive period) that has the most sand flux magnitude $\max\{\langle|\vec{q}|\rangle\}$ (kg/m/s). All plots have the same axes. Grey lines represent the vector $\vec{K}$ that makes the angles which are given in Figure 4b. Error bars denote ±1 standard deviation of ensemble members for all variables and sand seas. Vertical lines crossing each plot denote when the most active season in sand flux contributes to its equal share (1/4, left) and majority (1/2, right) of annual flux.

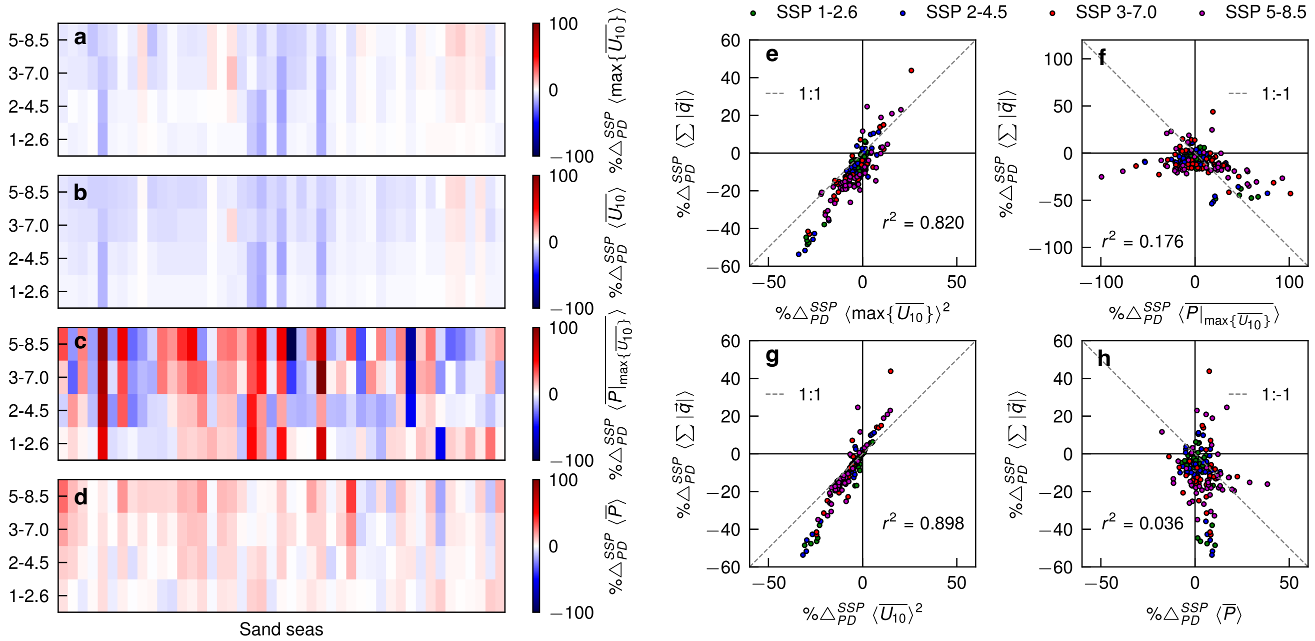

Figure S6: **Changes in wind speed and precipitation for sand seas.** Similarly to Figure 3a–e, (a–d) give the average (spatially across the sand sea, across ensemble members and in time across the decade) percentage relative change from the 2005-2014 historical decade to the 2091-2100 future decade in the four tier-1 SSP scenarios (vertical-axis in order of ascending radiative forcing) $\%\Delta_{PD}^{SSP}$ for each sand sea (horizontal-axis in order of descending area) in: (**a**) average 10-m wind speed during the maximum quarter $\langle \max\{\overline{U_{10}}\}\rangle$ (m/s), (**b**) annually-averaged 10-m wind speed $\langle\overline{U_{10}}\rangle$ (m/s), (**c**) average precipitation flux during the quarter of maximum average 10-m wind speed $\langle \overline{P|_{\max\{\overline{U_{10}}\}}}\rangle$ (kg/m^2/s), and (**d**) annually-averaged precipitation flux $\langle\overline{P}\rangle$. Wind changes are relatively weak compared to the other variables considered in this article, while precipitation changes during the season of maximal winds are highly variable because they are typically extremely arid seasons in sand seas. (**e**–**h**) show how the variables in (a–d), respectively, contribute to average (spatially across the sand sea, across ensemble members and in time across the decade) percentage relative changes in absolute sand flux magnitude from the 2005-2014 historical decade to the 2091-2100 future decade in the four tier-1 SSP scenarios (colors of dots in legend above panels) $\%\Delta_{PD}^{SSP}\langle\sum|\vec{q}|\rangle$ (kg/m/s). (e&f) show the square of wind speed, not wind speed, as this is the scaling with flux (this is why the relative change in flux is larger than wind, to first-order). Grey dashed lines show the expected first-order relationship between the variables, which holds well for the square of wind speed but not for precipitation, largely since precipitation doesn't occur mostly during winds in excess of threshold, especially during the transport season. Correlation coefficients between variables are given in (e–h), from which we see that changes in the season of strongest winds can explain almost as much variance in changes in annual sand flux magnitude as changes in annual winds, and that changes in precipitation—which mostly increases (weighting toward RHS of (h) or majority red in (d)—are essentially uncorrelated with changes in sand flux.

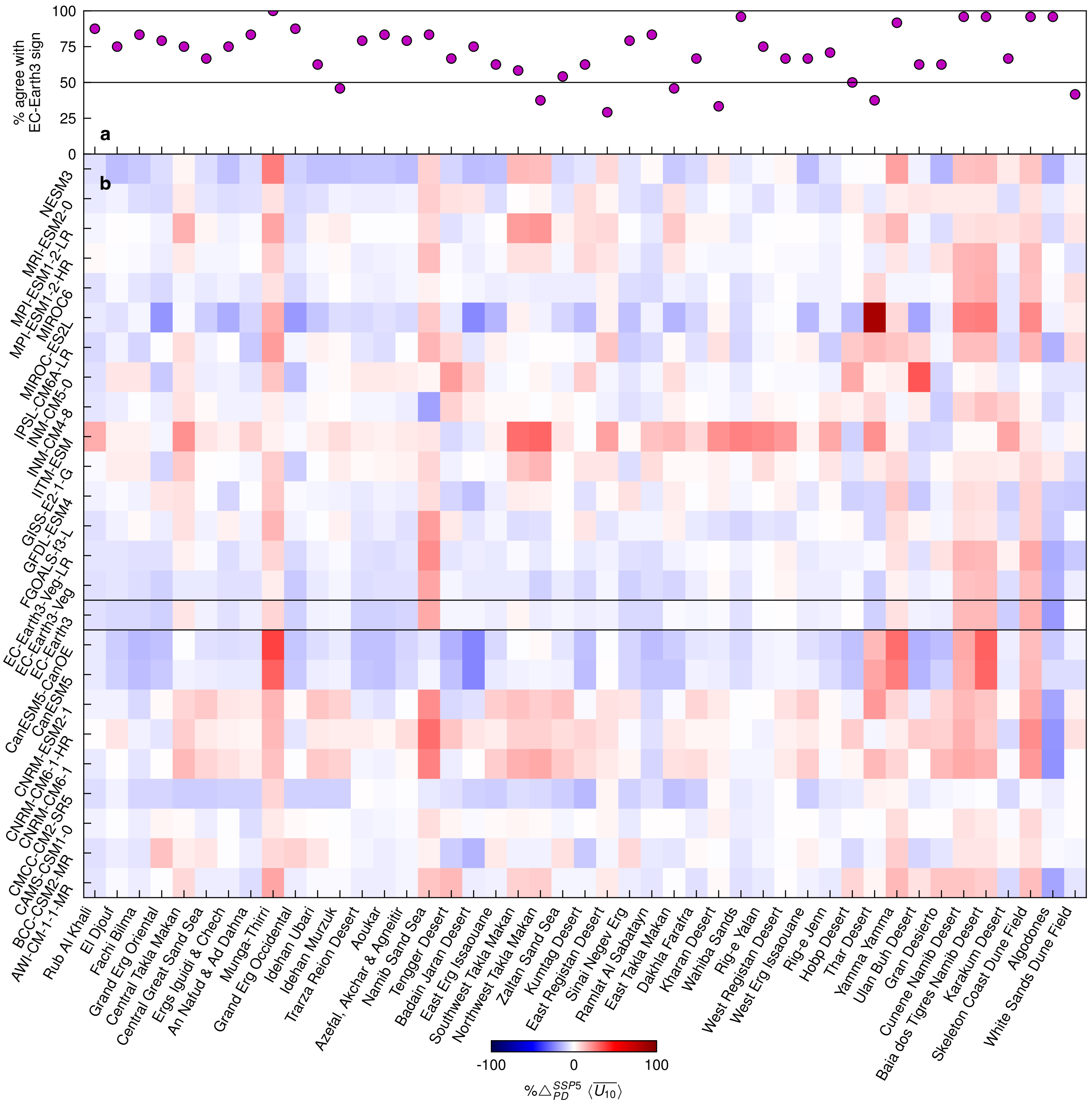

Figure S7: **Agreement of EC-Earth3 wind changes with other CMIP6 ESMs.** (**a**) Percentage of the 24 other CMIP6 ESMs that agree with the direction of average (spatially across the sand sea, across ensemble members and in time across the decade) change in 10-m wind speed $\langle\overline{U_{10}}\rangle$ (m/s) for each sand sea from the 2005-2014 decade to 2091-2100 decade in the highest radiative forcing SSP5-8.5 scenario. Majority agreement is noted by the horizontal 50% black line. (**b**) The percentage relative change in 10-m wind speed $\%\Delta_{PD}^{SSP5}\langle\overline{U_{10}}\rangle$ used to generate (a). Both panels share the horizontal axis, sand seas ordered in descending area order, while the vertical axis of (b) is the ESMs used in the comparison ordered alphabetically from the bottom. EC-Earth3 is bordered by black lines for clarity. Note that the magnitude of change in EC-Earth3 is not extreme comparatively and that sand seas in east Asia are those in least agreement with the other ESMs.

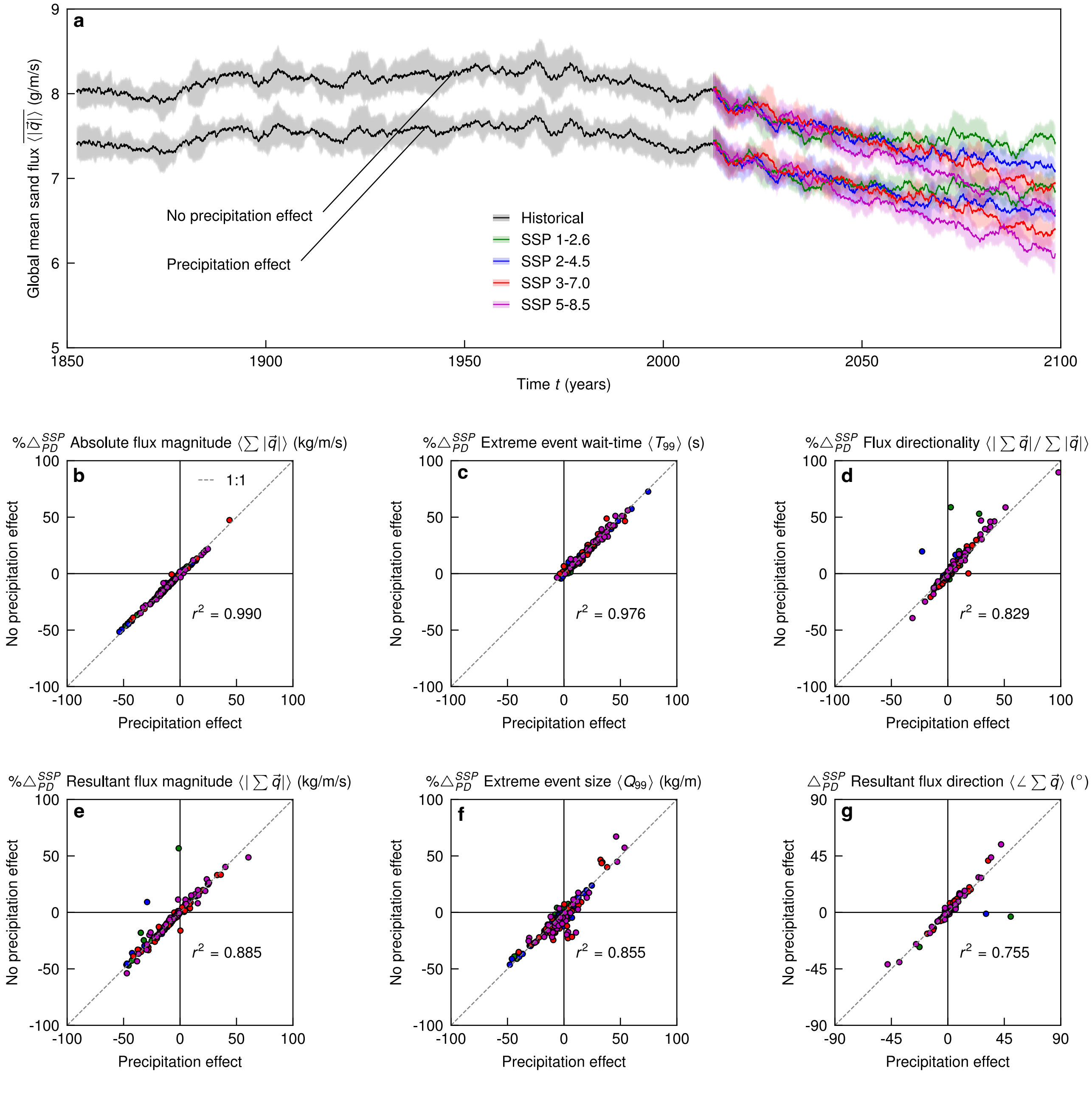

Figure S8: Caption on following page.

Figure S8: **The magnitude of the modeled role of precipitation in sand transport threshold.** In this work, we assume precipitation in-excess of a small value inhibits sand transport only over the 3-hour period it occurs. This is a first-order implementation; there are higher-order effects of precipitation on sand transport. To assess how important this first-order effect is, we compare it against predicted sand sea activity as if precipitation has zero effect. (**a**) Similarly to Figure 2a but for both with and without precipitation effect, we plot modelled time series of the 5-year smoothed globally-averaged sand flux magnitude $\overline{\langle|\vec{q}|\rangle}$ (g/m/s) for the historical (black) and future SSP (1-2.6, green; 2-4.5, blue; 3-7.0, red; 5-8.5, purple) scenarios; ensemble mean (lines) and ±1 standard deviation (shaded envelopes) are shown. Similarly to Figure 3a–f, (b–g; colors as in the legend of a) give the average (spatially across the sand sea, across ensemble members and in time across the decade) percentage (absolute for g) relative change from the 2005-2014 historical decade to the 2091-2100 future decade in the four tier-1 SSP scenarios $\%\Delta_{PD}^{SSP}$ for each sand sea with (horizontal axis) and without (vertical axis) the first-order influence of precipitation on sand flux in: (**b**) absolute flux magnitude $\langle\overline{|\vec{q}|}\rangle$ (kg/m/s), (**c**) 99th percentile flux event wait-time $\langle T_{99}\rangle$ (s), (**d**) flux directionality $\langle|\sum\vec{q}|/\sum|\vec{q}|\rangle$, (**e**) resultant flux magnitude $\langle|\sum\vec{q}|\rangle$ (kg/m/s), (**f**) 99th percentile flux event size $\langle Q_{99}\rangle$ (kg/m), (**g**) resultant flux direction $\angle\sum\vec{q}$ (°). It is clear that while neglecting the precipitation effect necessarily increases the overall flux (by 8.5% on average in the historical period), relative changes in sand sea activity over the century in a number of measures are similar with and without precipitation's first-order role (high r^2 values).

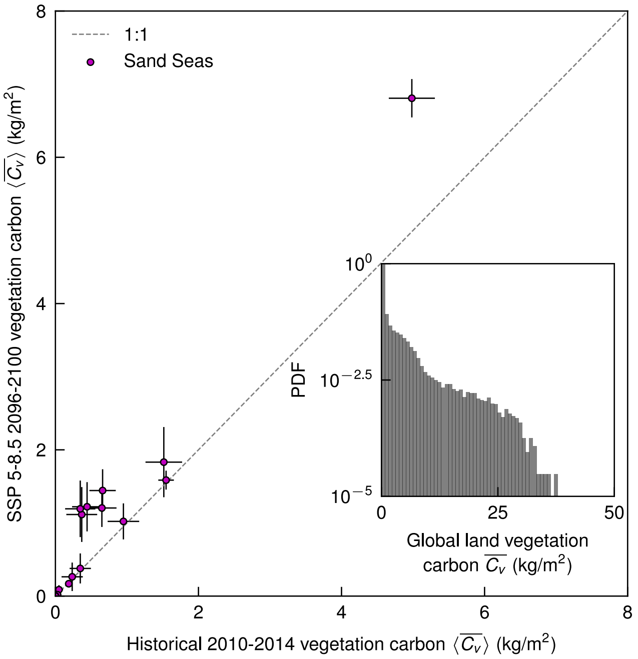

Figure S9: **Vegetation in the EC-Earth3Veg ESM.** In order to assess the role of vegetation in the sand seas studied here, we look to the EC-Earth3 ESM's counterpart model that includes an online vegetation module. In the main panel we plot the average (purple dots) and standard deviation (black lines) of monthly land vegetation carbon mass per area $\langle \overline{C_V} \rangle$ (kg/m^2) for each sand sea across 6 ensemble members during the 5-year period ending the historical (horizontal axis; i.e. present-day) and SSP 5-8.5 (vertical axis) scenarios. The dashed grey line denotes equal vegetation cover in scenarios. Generally vegetation cover in the sand seas is predicted to stay the same or increase modestly from present-day to the end of the century in the most severe SSP scenario. The magnitude of the vegetation cover in the sand seas is contextualized by the inset, the probability density function of global C_V in the present day; as expected, the sand seas studied here are predicted to have very low vegetation cover relative to the rest of the world (28 of 45 have $\langle \overline{C_V} \rangle = 0$).
